# Choosing Important Health Outcomes for Comparative Effectiveness Research: An Updated Review and User Survey

**DOI:** 10.1371/journal.pone.0146444

**Published:** 2016-01-19

**Authors:** Sarah L. Gorst, Elizabeth Gargon, Mike Clarke, Jane M. Blazeby, Douglas G. Altman, Paula R. Williamson

**Affiliations:** 1 Department of Biostatistics, University of Liverpool, Liverpool, United Kingdom; 2 Northern Ireland Network for Trials Methodology Research, Queen's University Belfast, Belfast, United Kingdom; 3 School of Social and Community Medicine, University of Bristol, and Division of Surgery, Head & Neck, University Hospitals NHS Foundation Trust, Bristol, United Kingdom; 4 Centre for Statistics in Medicine, University of Oxford, Oxford, United Kingdom; Mario Negri Institute for Pharmacology Research, ITALY

## Abstract

**Background:**

A COS represents an agreed minimum set of outcomes that should be measured and reported in all trials of a specific condition. The COMET (Core Outcome Measures in Effectiveness Trials) initiative aims to collate and stimulate the development and application of COS, by including data on relevant studies within a publically available internet-based resource. In recent years, there has been an interest in increasing the development of COS. Therefore, this study aimed to provide an update of a previous review, and examine the quality of development of COS. A further aim was to understand the reasons why individuals are searching the COMET database.

**Methods:**

A multi-faceted search strategy was followed, in order to identify studies that sought to determine which outcomes/domains to measure in clinical trials of a specific condition. Additionally, a pop up survey was added to the COMET website, to ascertain why people were searching the COMET database.

**Results:**

Thirty-two reports relating to 29 studies were eligible for inclusion in the review. There has been an improvement in the description of the scope of a COS and an increase in the proportion of studies using literature/systematic reviews and the Delphi technique. Clinical experts continue to be the most common group involved in developing COS, however patient and public involvement has increased. The pop-up survey revealed the most common reasons for visiting the COMET website to be thinking about developing a COS and planning a clinical trial.

**Conclusions:**

This update demonstrates that recent studies appear to have adopted a more structured approach towards COS development and public representation has increased. However, there remains a need for developers to adequately describe details about the scope of COS, and for greater public engagement. The COMET database appears to be a useful resource for both COS developers and users of COS.

## Introduction

When designing clinical trials, it is important to measure appropriate outcomes, so that the results can be compared with other trials and will be as useful as possible to decision makers. At present, many studies which explore the effects of the same intervention on a specific health condition measure and report different outcomes, making it difficult to compare, contrast or combine their findings. This causes problems for people trying to use healthcare research. Inconsistency in outcome measurement and outcome reporting bias have led to avoidable waste in the production and reporting of research [1,2]. However, these issues could be reduced through the development and application of core outcome sets (COS). A COS represents an agreed minimum set of outcomes that should be measured and reported in all trials of a specific condition [3]. The existence of a COS allows the results of trials to be brought together as appropriate and ensures that all trials provide usable evidence. COS are not intended to restrict the number of outcomes in a particular trial, rather, the intention is that the outcomes in the COS will always be collected and reported, and it is fully expected that researchers will continue to explore additional outcomes [3].

The COMET (Core Outcome Measures in Effectiveness Trials) initiative aims to collate and stimulate the development, application and promotion of COS, by including data on relevant individual studies within a publically available internet-based resource. The COMET database is a unique inventory that includes published accounts of COS development, as well as planned and ongoing work. Initially, the database included studies that had been identified by ad hoc means and a systematic review which addressed outcome selection in clinical trials in children [4]. In order to bring the database content up-to-date and be comprehensive, a systematic approach was needed to identify relevant material. Gargon and colleagues [5] conducted a systematic review in 2013, the first comprehensive search for COS in health research, and identified 198 studies that determined which outcomes or domains should be measured in all clinical trials for a specific health condition. The review revealed wide variation in the methods used to develop COS, and highlighted the need for methodological guidance, including how to engage key stakeholder groups, particularly members of the public, in the development and implementation of COS. When using the term ‘public’ through this report we include patients, carers, health and social care service users and people from organisations who represent these groups [6]. The involvement of the public in the development of COS is particularly relevant for comparative effectiveness research where long term patient centred outcomes are often the important endpoints.

Awareness of the need for COS and knowledge of the COMET Initiative has continued to grow, reflected in website and database usage figures [7]. More than 16,500 visits were made to the website in 2014 (36% increase over 2013) and 9780 new visitors (43% increase). By December 2014, a total of 6588 searches of the COMET database had been completed; however no information had been collected about the reasons for searching the database.

### Aims

To update the original systematic review [5], in order to identify any further studies where a COS has been developed, and to describe the methodological techniques used in these studies. In addition, to understand the reasons why individuals are searching the COMET database.

## Methods

### Systematic review update

The methods used in this updated review followed the same approach used in the original review [5].

#### Study selection. Inclusion and exclusion criteria

As described in detail previously [5]. Broadly speaking, studies were eligible for inclusion if they had applied methodology for determining which outcome domains or outcomes should be measured, or are important to measure, in clinical trials or other forms of health research.

#### Types of participants and interventions

As previously [5], studies were categorised as eligible if they related to participants of any age, with any health condition, in any setting, and assessed the effect of any or all interventions for that condition.

#### Identification of relevant studies

In January 2015, we searched MEDLINE via Ovid and SCOPUS (including EMBASE) without language restrictions. The search identified studies that had been published from January 2013 onwards (MEDLINE) and August 2013 onwards (SCOPUS). The multifaceted search strategy developed in the original review using a combination of text words and index terms [8] was used in the current review, with adaptations appropriate for each database.

#### Selecting studies for inclusion in the review

Records from each database were combined and duplicates were removed. Titles and abstracts were read to assess eligibility of studies for inclusion in the review (stage 1). Full texts of potentially relevant articles were obtained to assess for inclusion (stage 2). Two of three reviewers (SG, JW and BG) independently checked the title and abstract of each citation. One reviewer (SG) assessed the title and abstract of all citations. A second reviewer (JW) assessed the title and abstract of the first half of the citations and a third reviewer (BG) assessed the title and abstract of the second half of the citations. Citations were retained for further checking if agreement could not be reached. One reviewer (SG) assessed each full paper for inclusion in the review and another reviewer (BG) assessed half of the full papers. Reasons for exclusion at this stage were documented for articles judged to be ineligible.

#### Checking for agreement between reviewers

During each stage of the review process, agreement between reviewers was assessed. Prior to independently assessing records, the three reviewers (SG, JW and BG) independently checked batches of abstracts and full papers for agreement.

#### Checking for correct exclusion

Of the records that had been excluded on the basis of the title and abstract, full text papers were obtained for a 1% sample and a fourth reviewer (EG) assessed correct exclusion. If any studies were identified as being incorrectly excluded, further checking was performed within the other excluded records. Of the records that had been excluded after reading their full text papers, 5% were assessed for correct exclusion at that stage.

#### Data extraction

As described in detail previously [5], data were extracted in relation to the study aims, health area, target population, methods of COS development and stakeholder groups involved.

#### Data analysis and presentation of results

As described previously [5], the results are presented descriptively.

### Pop-up survey

During May-June 2015, a pop up survey consisting of one question was added to the COMET website search page, with the intention of finding out why people were searching in the COMET database. At the beginning of each search, the survey would appear, to ask people to select a response as to their reason for searching in the COMET database. The survey was designed so that it was just one question with a set of multiple-choice answers, which was fully contained within the pop-window. This meant that people could answer the question and close the window with a single click. People were asked only once to do this although they could have run a number of searches.

## Results

### Description of studies

**Fig 1:**
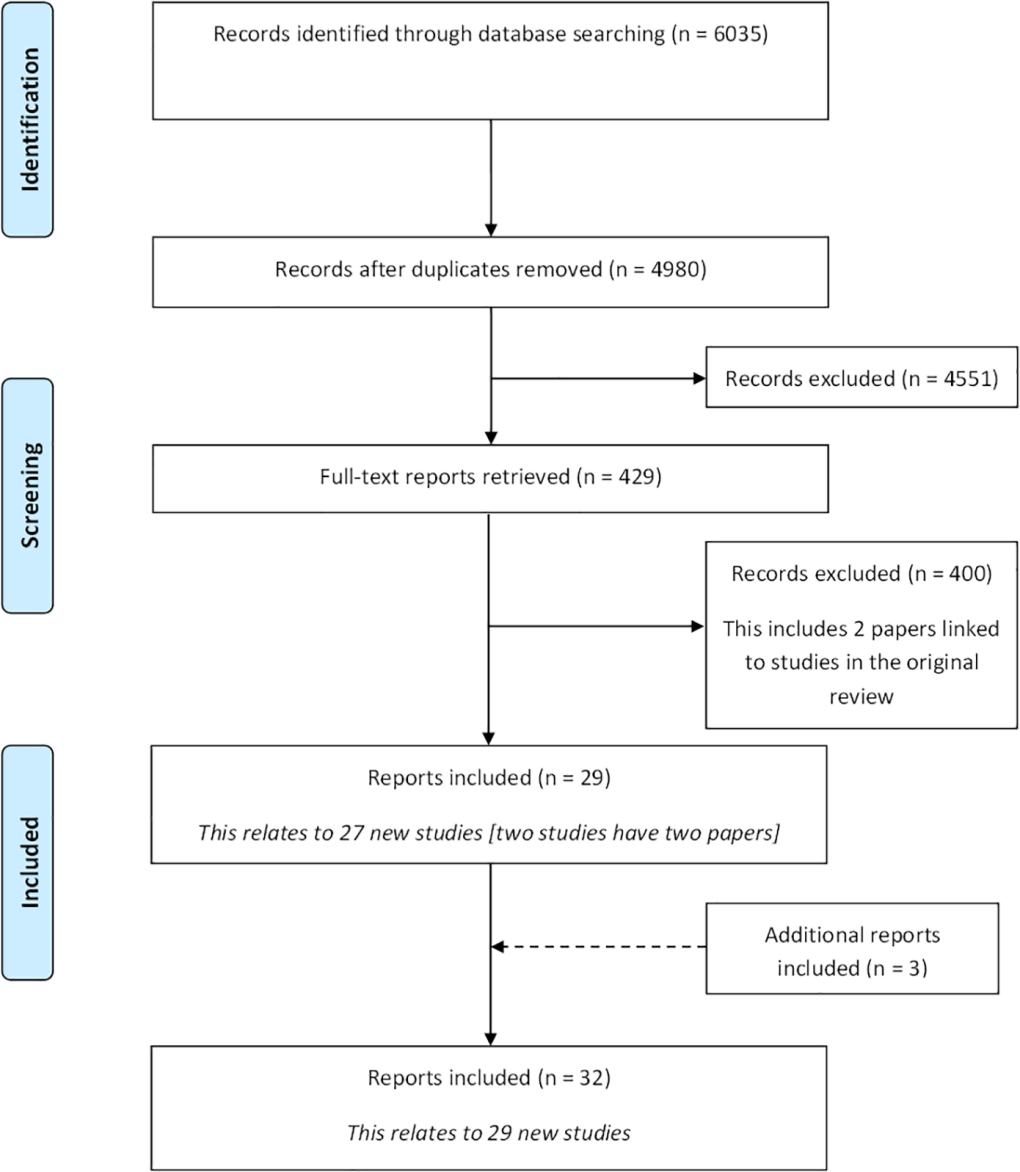
Identification of studies.

Following the removal of duplicates, 4980 citations were identified in the initial database search. A total of 4551 records were excluded during the title and abstract stage, and a further 400 were excluded following the assessment of full text papers (Fig 1). S1 Table provides a summary of the reasons for exclusion of the full text papers. Twenty-nine citations met the inclusion criteria. In addition to the database search, three additional citations were identified as being eligible for inclusion in the review following reference checking. In total, 32 reports relating to 29 new studies were included (S2 Table).

### Included studies

#### Year of publication

**Fig 2:**
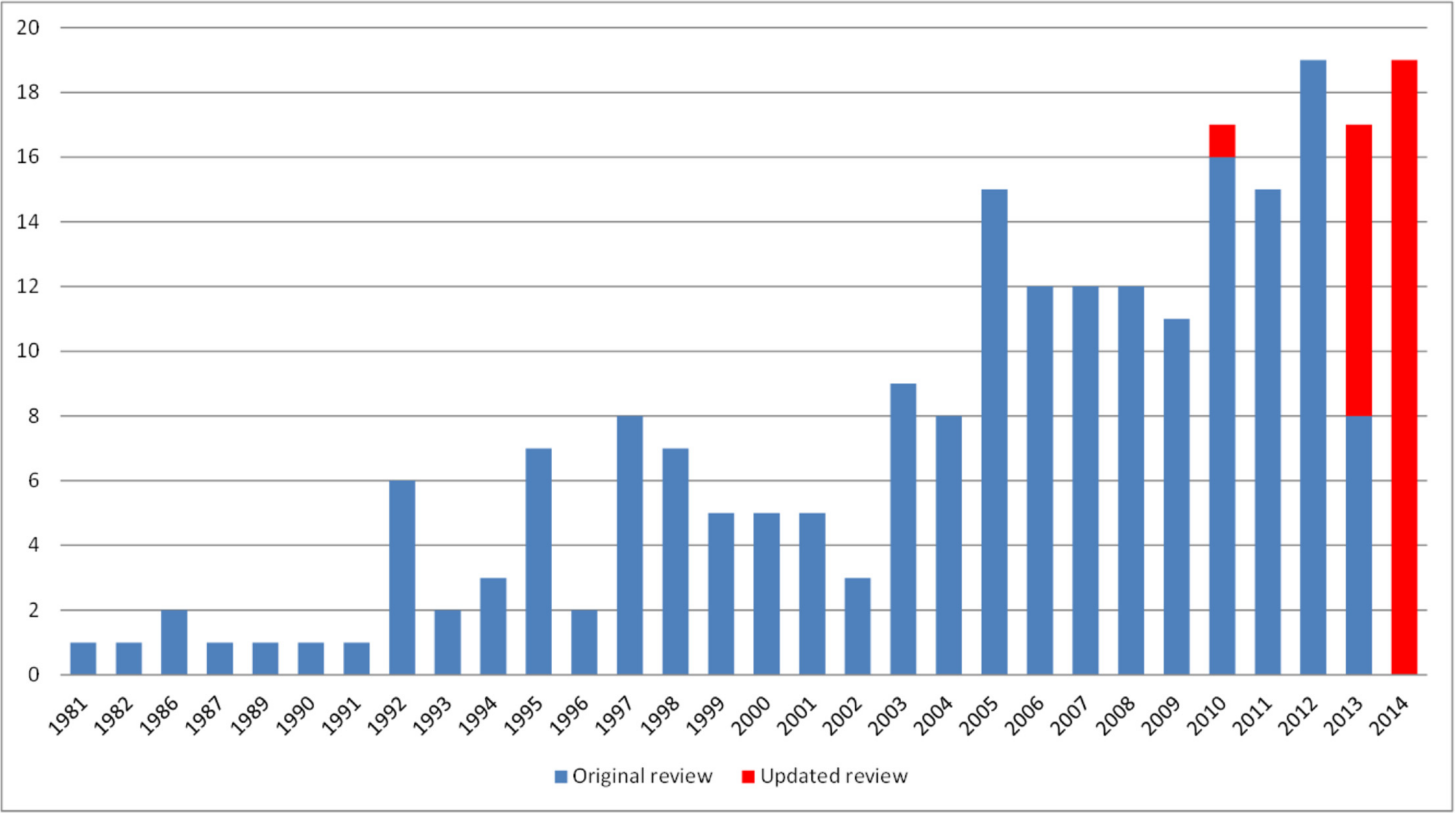
Year of first publication of each COS study (n = 227).

The figure displaying the year of first publication of each COS that was included in the original review has been updated to include the 29 new studies identified in this updated review (Fig 2). The figure confirms that there has been a general increase in the number of COS over the years, and shows that there has been a consistently higher number of COS published annually in recent years than in most years before 2010. Of the 29 studies identified in this update, 28 studies were published between 2013 and 2014, and one study was published in 2010. This study was identified in the searches for the original review [5], but the lack of information on the COS in the title and abstract meant that it was excluded in the initial stages of that review. This study was identified in the updated review, through the checking of references in the included studies, thus highlighting the value of checking reference lists for locating studies in systematic reviews [9].

#### Scope of core outcome sets

**Fig 3:**
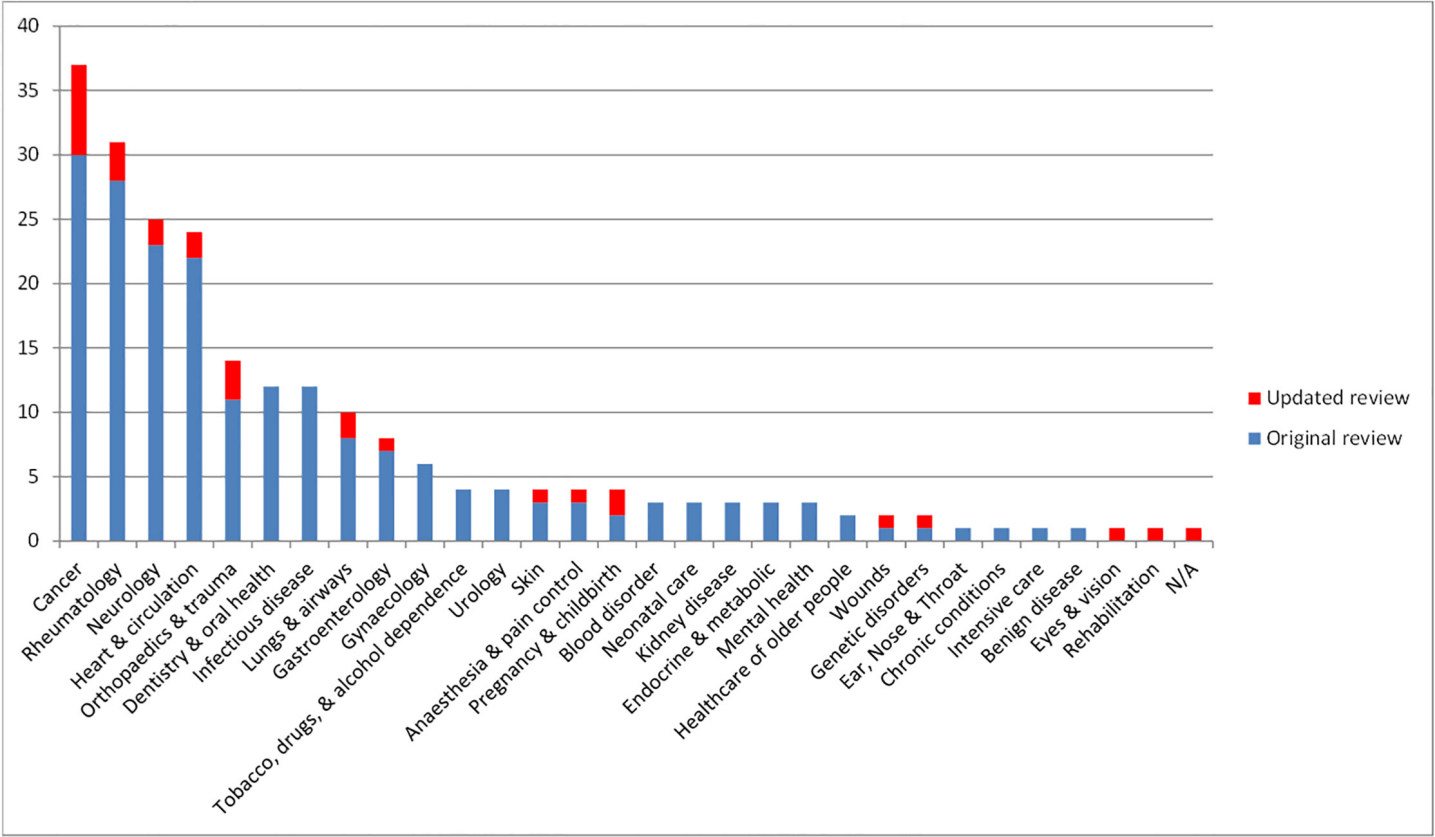
Number of COS developed in each disease category (n = 227).

Fig 3 shows the number of COS developed according to disease category. The classification of 227 published COS studies are presented in S3 Table. The scope of published COS studies is summarised in Table 1 and includes both the 198 COS included in the original review and the 29 new COS that have been added by this updated review. This includes study aims, setting for intended use, population characteristics and intervention characteristics.

**Table 1:** The scope of included studies (n = 227).

	Original review n (%)	Updated review n (%)	Combined n (%)
**Study aims**			
Specifically considered outcome selection and measurement	97 (49)	22 (76)	119 (52%)
Considered outcomes while addressing wider clinical trial design issues	101 (51)	7 (24)	108 (48%)
**Intended use of recommendations**			
Clinical trials	141 (71)	19 (66)	160 (71)
Clinical research	27 (14)	4 (14)	31 (14)
Clinical research and practice	11 (6)	4 (14)	15 (7)
Clinical trials and clinical practice	10 (5)	0 (0)	10 (4)
Clinical trials and regulatory purposes	3 (2)	0 (0)	3 (1)
Trials and observational studies	3 (2)	0 (0)	3 (1)
Clinical trial extension studies		1 (4)	1 (<1)
Clinical trials, research and clinical record keeping		1 (4)	1 (<1)
Observational studies	1 (<1)	0 (0)	1 (<1)
Trials and case series	1 (<1)	0 (0)	1 (<1)
Clinical research, clinical practice and regulatory purpose	1 (<1)	0 (0)	1 (<1)
**Population characteristics**			
Adults	10 (5)	11 (38)	21 (9)
Children	23 (12)	2 (7)	25 (11)
Adults and children	13 (7)	1 (4)	14 (6)
Older adults	3 (2)	1 (4)	4 (2)
Adults and neonates	0 (0)	1 (4)	1 (<1)
Not specified	149 (75)	13 (45)	162 (71)
**Intervention characteristics**			
All intervention types	7 (4)	9 (31)	16 (7)
Drug treatments	40 (20)	4 (14)	44 (19)
Surgery	13 (7)	4 (14)	17 (8)
*Surgery only*	*13*	*2*	
*Surgery and compression therapy*	*0*	*1*	
*Surgery and injection*	*0*	*1*	
Vaccine	2 (1)	0 (0)	2 (1)
Rehabilitation	1 (1)	1 (4)	2 (1)
Exercise	1 (1)	1 (4)	2 (1)
*Exercise (physical activity)*	*1*	*0*	
*Exercise (yoga)*	*0*	*1*	
Procedure*	5 (3)	0 (0)	5 (2)
Device**	3 (2)	0 (0)	3 (1)
Other***	11 (6)	5 (17)	16 (7)
Not specified	115 (58)	5 (17)	120 (53)

*Procedure descriptions–

*Procedure—Uterine artery embolization*.

*Procedure—Aortic valve stenosis (AS)—transcatheter aortic valve implantation*.

*Procedure—Aortic valve stenosis (AS)*.

*Procedure—pulp treatments of primary teeth*.

*Procedure—drug-eluting coronary stents (DES)*.

**Device descriptions–

*Device–Compression (n = 2)*.

*Device—Mechanical circulatory support (MCS)*.

***Other descriptions–

Coronary angiogenesis.

Hip protectors.

Neuro-protective therapy (aka Neuroprotection).

Non-surgical treatment (no other detail given).

Operative and non-operative management.

Oral care products.

Ascorbic acid.

Fall injury prevention interventions.

Behavioural therapies or other kinds of nonpharmacologic therapies.

Psychological & behavioural: Psychosocial.

Maternity care.

*Complementary and alternative medicine (CAM)*.

*Chemotherapy*.

*Platelet transfusion trials*.

*Endovascular therapy*.

*Constraint-induced movement therapy (CIMT)*.

There has been an improvement in the description of the scope of a COS. In the original review, only 25% (49/198) of studies were found to clearly define the population characteristics, as compared to 55% (n = 16/29) of studies in the updated review. The majority, 58% (n = 115/198), of studies in the original review neglected to report intervention characteristics, compared to 17% (n = 5/29) in the update.

#### Methods used to select outcomes

COS have been developed using a variety of methods [5]. The inclusion of a literature/systematic review to inform the development has increased from 33% (n = 66/198) of studies in the original review to 72% (n = 21/29) of studies in the updated review. The use of the Delphi technique for assessing and developing consensus has risen from 15% (n = 29/198) in the original review to 31% (n = 9/29) in the updated review.

#### People involved in selecting outcomes

The stakeholder groups regarded as key to developing a COS will vary between clinical areas. Clinical experts continue to be involved in almost all studies (see S4 Table), but there has been a trend towards greater involvement of patient and public representatives, increasing from 18% (31/174) in the original review, to 59% (13/22) in this updated review. Table 2 describes the degree of patient representation reported in COS studies included in this updated review.

**Table 2:** Public involvement detail where reported (n = 6).

	Methods used	Total number of participants	Number of public participants	% Public participants
1	Meeting and teleconferences (mixed)	6	1	17%
2	Nominal Group Technique (mixed)	25	3	12%
3	Meeting (mixed)	12	1	8%
4	Focus groups (patient only)	45	45	
	Delphi (clinician only)	Round 1: 249	Round 1: 249	
		Round 2: 247	Round 2: 247	
		Round 3: 247	Round 3: 247	
	Nominal Group Technique (mixed)	28	5	18%
5*	Delphi (patient only)	Round 1: 169	Round 1: 169	
		Round 2: 152	Round 2: 152	
		Round 3: 147	Round 3: 147	
6	Delphi (patient only)	Round 1: 71	Round 1: 71	
		Round 2: 67	Round 2: 67	
		Round 3: 62	Round 3: 62	
	Delphi (clinician only)	Round 1: 39	Round 1: 39	
		Round 2: 35	Round 2: 35	
		Round 3: 33	Round 2: 33	
		Round 2: 35	Round 2: 35	
		Round 3: 33	Round 2: 33	

* *Patient core set*

### Pop-up survey

During the four-week period of the survey, 396 different people searched the database. The survey achieved a 52% (206/396) response rate. Table 3 shows the frequency of the reasons selected. The most common were ‘I am thinking about developing a core outcome set’ and ‘I am planning a clinical trial’.

**Table 3:** Results from pop-up survey.

Reason	N
I am thinking about developing a core outcome set	49
We are considering funding a core outcome set	3
I am reviewing a funding application for the development of a core outcome set	0
I have been asked to take part in a core outcome set study	2
I am a person with a condition	0
I am planning a systematic review of clinical trials	19
I am planning a clinical trial	33
I am reviewing a funding application for a trial	4
We are considering funding a clinical trial	2
I am involved in auditing a condition	6
As part of a general educational activity	29
General interest	27
None of the above	32
Did not respond	190
**Total**	396

## Discussion

As in the original review, studies identified in this update covered various areas of health, with cancer and rheumatology being the most common. In addition to identifying the areas where COS are continuing to be developed, the review identified that COS had been developed for the first time in the areas of rehabilitation and eyes & vision. Regular review updates will allow us to identify the areas where there is an ongoing absence of COS, which may highlight future opportunities for COS developers.

It is apparent that many studies still do not report important characteristics pertaining to the scope of the COS. The development and implementation of a reporting guideline, may facilitate improved reporting of COS, a project currently underway [10].

A variety of methods continue to be used to develop COS. However, there has been an increase in the proportion of studies using literature/systematic reviews and the Delphi technique. As in the original review, the updated review found clinical experts to be the most common group involved in developing COS. However, the proportion of studies that reported patient and public involvement in the process has increased. Since the systematic search was completed in January 2015, we are aware of a further six COS studies that have been published, and of these six studies, 100% have included patient and public representatives. In addition, patient and public representatives are also involved in 89% (64/72) of the planned and ongoing studies registered in the COMET database. It is important that clinical trials include outcomes that are considered to be important to patients and other users of health and social care. It appears that the issue of patient and public involvement is no longer about whether to involve, but rather how. In response to this need, COMET has set up the PoPPIE (People and Patient Participation, Involvement and Engagement) Working Group to develop resources in this area. As an example, plain language summaries are being developed in partnership with patients, http://www.comet-initiative.org/resources/PlainLanguageSummary, and a research agenda around methods for patient engagement has been set.

### Implications

The studies identified in this updated review have been added to the COMET database; a freely accessible, publically available, searchable online resource that shows what work has been done in relation to outcomes to measure in effectiveness trials. This update has ensured that the database stores the most current research, and provides readily available information about existing COS, which will assist researchers, practitioners and other stakeholders, including the public, when designing new clinical trials, systematic reviews and clinical guidelines. Moreover, it will also help to avoid unnecessary duplication of efforts in COS development.

The results of the pop up survey demonstrate that people thinking about developing a COS are checking the COMET database to see whether a COS exists in their area of interest to avoid such duplication, hence emphasising the importance of keeping the database current. The pop-up survey also shows that the COMET database is being used for a variety of reasons by people besides COS developers, including clinical trialists, systematic reviewers, auditors, COS funders, and people who have been asked to participate in the development of COS. Interestingly, nearly one sixth of respondents were searching for reasons not anticipated, and further research is needed to understand those. Although 48% of visitors to the website did not respond to the survey, closing down the pop-up box immediately, this may reflect a typical response rate for such surveys.

### Limitations

There are a number of limitations of this review that should be noted. First, although two well-established, large bibliographic databases were searched to identify studies meeting the inclusion criteria, we may have missed some relevant studies within these databases, either because the reports of these studies are not indexed in these databases or because the information in the indexed record is insufficient to show that the report is eligible. It would be beneficial for bibliographic databases to introduce an indexing term to facilitate easier identification of COS. As shown by both our original review [8] and this update, the identification of COS is a complex task and we will keep the search strategy under review and refine it as necessary to minimise the possibility of missing eligible studies in the future.

A second limitation of the review is that the lack of an assessment tool means that there has been no formal quality assessment of the included studies. Determining the quality of a COS is not straightforward. A high quality COS would be one that leads to improved health outcomes but this would be difficult to measure. A tool is needed to assess how the COS developers minimised biases which would otherwise undermine the ability of the COS to have a positive impact on patient care and outcomes.

### Conclusion

We have provided an updated review of studies that have addressed the development of COS for measurement and reporting in clinical trials. The updated review has demonstrated that COS are continuing to be developed across a range of health areas. Recent studies appear to have adopted (or, at least, reported) a more structured approach towards COS development, and public representation has increased. However, although general reporting quality has increased, there remains a need for developers to adequately describe details about the scope of COS, and for greater public engagement. The COMET database is likely to be a useful resource for both COS developers and users of COS including clinical trialists and systematic reviewers.

## Supporting Information

S1 PRISMA Checklist PRISMA checklist for content of a systematic review. (DOC)

S1 Table Reason for exclusion at stage 2 (assessment of full text reports). (DOCX)

S2 Table Table of reports included in updated review (n = 32). (DOCX)

S3 Table Classification of COS according to condition (n = 227 published studies). (DOCX)

S4 Table Participant groups involved in selecting outcomes in new studies identified in the review update (n = 29). (DOCX)
