# Supplementary material for: Choosing Important Health Outcomes for Comparative Effectiveness Research: An Updated Review and User Survey
Source: PLoS One. 2016 Jan 19;11(1):e0146444. doi: 10.1371/journal.pone.0146444 (PMC4718543; doi:10.1371/journal.pone.0146444)
Supplement: S1 Table — (DOCX) [file pone.0146444.s002.docx]

**S1 Table.** Reason for exclusion at stage 2 (assessment of full text reports): Categories are as reported in original review

| **Reason** | **n** |
| --- | --- |
| Review/overview/discussion only, no outcome recommendations | 94 |
| Core outcomes/ outcome recommendations not made | 24 |
| Health-related quality of life (HRQL)* | 5 |
| Studies relating to how, rather than which, outcomes should be measured | 56 |
| ICF core set development | 0 |
| Quality indicators – included an aspect of outcomes | 0 |
| Not relevant | 133 |
| ICF core set validation | 0 |
| Quality indicators – structure and/or process of care only | 2 |
| One outcome/ domain only | 7 |
| Recommendations for clinical management in practice not research | 38 |
| Instrument development | 2 |
| Recommendations by single author only | 1 |
| Registry development | 0 |
| Describes features of registry | 0 |
| Preclinical/ Early phase only (0, I, II) | 2 |
| Ongoing studies | 9 |
| Duplicate | 11 |
| Quantitative description (e.g. frequency of symptoms) | 0 |
| Studies reporting the design/ rationale of single trial | 1 |
| Oral presentation only | 0 |
| Value attributed to outcomes | 1 |
| Studies reporting the use of a COS** | 1 |
| Systematic reviews of clinical trials** | 2 |
| Studies that elicit stakeholder group opinion regarding which outcome domains or outcomes are important** | 6 |
| Assessed in original review** | 14 |
| Linked to studies published in original review** | 2 |
| **TOTAL** | **400** |

** These studies included qualitative studies describing the impact of a treatment on a patient’s quality of life, studies to determine particular domains of quality of life, and single patient narratives of the impact of a condition or treatment on their quality of life. The focus of these studies was quality of life only.*

*** These studies did not fit into any of the existing categories identified in the original review and so additional categories were created.*
