# Supplementary material for: Choosing Important Health Outcomes for Comparative Effectiveness Research: An Updated Review and User Survey
Source: PLoS One. 2016 Jan 19;11(1):e0146444. doi: 10.1371/journal.pone.0146444 (PMC4718543; doi:10.1371/journal.pone.0146444)
Supplement: S2 Table — (DOCX) [file pone.0146444.s003.docx]

**S2 Table.** Table of reports included in updated review (n=32)

| **Study** | **Disease category** | **Disease name** |
| --- | --- | --- |
| Chen 2014 [1]** | Cancer | Prostate cancer |
| Chera 2014 [2]** | Cancer | Head and neck cancer |
| Fraser 2013 [3]** | Cancer | Head and neck cancer |
| Donovan 2014 [4]** | Cancer | Ovarian cancer |
| Glynne-Jones 2014 [5]** | Cancer | Anal cancer |
| Reeve 2014 [6]** | Cancer | Cancer/Malignant disease |
| Wildiers 2013 [7]** | Cancer | Cancer/Malignant disease |
| Buch 2014 [8]* | Rheumatology | Rheumatic disease |
| Ward 2014 [9]* | Rheumatology | Rheumatic disease |
| Kloppenburg 2014 [10]** | Rheumatology | Hand osteoarthritis |
| Ball 2013 [11]** | Orthopaedics & trauma | Dupuytren’s disease |
| Goldhahn 2014 [12]** | Orthopaedics & trauma | Distal radius fractures |
| Haywood 2014 [13]** | Orthopaedics & trauma | Hip fracture |
| Saketkoo 2014 [14]**  Saketkoo 2014 [15] | Lungs & airways | connective tissue disease associated interstitial lung disease (CTD-ILD) and idiopathic pulmonary fibrosis (IPF) |
| Spragg 2010 [16]* | Lungs & airways | Acute lung injury (ALI) |
| Eliasson 2014 [17]* | Neurology | Unilateral cerebral palsy |
| Smelt 2014 [18]** | Neurology | Migraine |
| Fong 2014 [19]** | Pregnancy & childbirth | Pre-eclampsia |
| Smith 2014 [20]* | Pregnancy & childbirth | Breech presentation |
| Diehm 2013 [21]** | Heart & circulation | Aortic dissection |
| Zannad 2013 [22]** | Heart & circulation | Heart failure |
| Plotkin 2013 [23]**  Wolters 2013 [24] | Genetic disorders | Neurofibromatosis |
| Ruemmele 2014 [25]** | Gastroenterology | Inflammatory bowel disease (IBD) |
| Samuel 2013 [26]* | Wounds | Venous leg ulcers |
| Simpson 2013 [27]** | Skin | Vulval skin condition |
| Wylde 2014 [28]** | Anaesthesia & pain control | Chronic post-surgical pain after total knee replacement |
| Feldman 2014 [29]**  Neville 2014 [30] | Rehabilitation | Recovery after surgery |
| Chiu 2014 [31]** | Eyes & vision | Intermittent exotropia |
| Cook 2013 [32]* | Other | Platelet transfusion trials |

** Considered outcomes while addressing wider clinical trial design issues*

*** Specifically considered outcome selection and measurement*

**References**

1. Chen RC, Chang P, Vetter RJ, Lukka H, Stokes WA, Sanda MG, et al. Recommended patient-reported core set of symptoms to measure in prostate cancer treatment trials. J Natl Cancer Inst. 2014;106(7).
2. Chera BS, Eisbruch A, Murphy BA, Ridge JA, Gavin P, Reeve BB, et al. Recommended patient-reported core set of symptoms to measure in head and neck cancer treatment trials. J Natl Cancer Inst. 2014;106(7).
3. Fraser JF, Hussain MS, Eskey C, Abruzzo T, Bulsara K, English J, et al. Reporting standards for endovascular chemotherapy of head, neck and CNS tumors. J Neurointerv Surg. 2013;5(5):396-399.
4. Donovan KA, Donovan HS, Cella D, Gaines ME, Penson RT, Plaxe SC, et al., Recommended patient-reported core set of symptoms and quality-of-life domains to measure in ovarian cancer treatment trials. J Natl Cancer Inst. 2014;106(7).
5. Glynne-Jones R, Adams RA, Jitlal M, Meadows H. End points in anal cancer: hopes for a common language. J Clin Oncol. 2014;32(12):1281-1282.
6. Reeve BB, Mitchell SA, Dueck AC, Basch E, Cella D, Reilly CM, et al. Recommended patient-reported core set of symptoms to measure in adult cancer treatment trials. J Natl Cancer Inst. 2014;106(7).
7. Wildiers H, Mauer M, Pallis A, Hurria A, Mohile SG, Luciani A, et al. End points and trial design in geriatric oncology research: a joint European organisation for research and treatment of cancer--Alliance for Clinical Trials in Oncology--International Society Of Geriatric Oncology position article. J Clin Onco. 2013;31(29):3711-3718.
8. Buch MH, Silva-Fernandez L, Carmona L, Aletah D, Christensen R, Combe B, et al. Development of EULAR recommendations for the reporting of clinical trial extension studies in rheumatology. Ann Rheum Dis. 2014
9. Ward L, Stebbings S, Sherman KJ, Cherkin D, Baxter GD, et al. Establishing key components of yoga interventions for musculoskeletal conditions: a Delphi survey. BMC Complement Altern Med. 2014;14:196.
10. Kloppenburg M, Bøyesen P, Smeets W, Haugen IK, Liu R, Visser W, et al. Report from the omeract hand osteoarthritis special interest group: Advances and future research priorities. Rheumatol. 2014;41(4):810-818.
11. Ball C, Pratt AL, Nanchahal J. Optimal functional outcome measures for assessing treatment for Dupuytren’s disease: a systematic review and recommendations for future practice. BMC Musculoskelet Disord. 2013;14:131.
12. Goldhahn J, Beaton D, Ladd A, Macdermid J, Hoang-Kim A; Distal Radius Working Group of the International Society for Fracture Repair (ISFR); International Osteoporosis Foundation (IOF). Recommendation for measuring clinical outcome in distal radius fractures: a core set of domains for standardized reporting in clinical practice and research. Arch Orthop Trauma Surg. 2014;134(2):197-205.
13. Haywood KL, Griffin XL, Achten J, Costa ML. Developing a core outcome set for hip fracture trials. Bone Joint J. 2014;96-B(8):1016-1023.
14. Saketkoo LA, Mittoo S, Frankel S, LeSage D, Saver C, Phillips K, et al. Reconciling healthcare professional and patient perspectives in the development of disease activity and response criteria in connective tissue disease-related interstitial lung diseases. J Rheumatol. 2014;41(4):792-798.
15. Saketkoo LA, Mittoo S, Huscher D, Khanna D, Dellaripa PF, Distler O, et al. Connective tissue disease related interstitial lung diseases and idiopathic pulmonary fibrosis: provisional core sets of domains and instruments for use in clinical trials. Thorax. 2014;69(5): 428-436.
16. Spragg RG, Bernard GR, Checkley W, Curtis JR, Gajic O, Guyatt G, et al. Beyond Mortality - Future Clinical Research in Acute Lung Injury. Am J Respir Crit Care Med. 2010;181(10): 1121-1127.
17. Eliasson AC, Krumlinde-Sundholm L, Gordon AM, Feys H, Klingels K, Aarts PB, et al. Guidelines for future research in constraint-induced movement therapy for children with unilateral cerebral palsy: an expert consensus. Dev Med Child Neurol. 2014;56(2):125-137.
18. Smelt AF, Louter MA, Kies D, Blom J, Terwindt G, van der Heijden GJ, et al. What do patients consider to be the most important outcomes for effectiveness studies on migraine treatment? Results of a Delphi study. PLoS One. 2014;6;9(6):e98933.
19. Fong F, Rogozinska E, Allotey J, Kempley S, Shah DK, Thangaratinam S., et al. Development of maternal and neonatal composite outcomes for trials evaluating management of late-onset pre-eclampsia. Hypertens Pregnancy. 2014;33(2):115-131.
20. Smith CA, Betts D. The practice of acupuncture and moxibustion to promote cephalic version for women with a breech presentation: implications for clinical practice and research. Complement Ther Med. 2014;22(1):75-80.
21. Diehm N, Vermassen F, van Sambeek MR; DEFINE Investigators. Standardized definitions and clinical endpoints in trials investigating endovascular repair of aortic dissections. Eur J Vasc Endovasc Surg. 2013;46(6):645-650.
22. Zannad F, Garcia AA, Anker SD, Armstrong PW, Calvo G, Cleland JG, et al. Clinical outcome endpoints in heart failure trials: a European Society of Cardiology Heart Failure Association consensus document. Eur J Heart Fail. 2013;15(10): 1082-1094.
23. Plotkin SR, Blakeley JO, Dombi E, Fisher MJ, Hanemann CO, Walsh KS, et al. Achieving consensus for clinical trials: the REiNS International Collaboration. Neurology. 2013;81(21 Suppl 1):S1-5.
24. Wolters PL, Martin S, Merker VL, Gardner KL, Hingtgen CM, Tonsgard JH, et al. Patient-reported outcomes in neurofibromatosis and schwannomatosis clinical trials. Neurology. 2013;81(21 Suppl 1):S6-14.
25. Ruemmele FM, Hyams JS, Otley A, Griffiths A, Kolho KL, Dias JA, et al. Outcome measures for clinical trials in paediatric IBD: An evidence-based, expert-driven practical statement paper of the paediatric ECCO committee. Gut. 2015;64(3):438-46.
26. Samuel N, Carradice D, Wallace T, Smith GE, Chetter IC. Endovenous thermal ablation for healing venous ulcers and preventing recurrence. Cochrane Database Syst Rev. 2013;10:CD009494.
27. Simpson RC, Thomas KS, Murphy R. Outcome measures for vulval skin conditions: a systematic review of randomized controlled trials. Br J Dermatol. 2013;169(3):494-501.
28. Wylde V, MacKichan F, Bruce J, Gooberman-Hill R. Assessment of chronic post-surgical pain after knee replacement: Development of a core outcome set. Eur J Pain. 2015;19(5):611-20.
29. Feldman LS, Lee L, Fiore J Jr. What outcomes are important in the assessment of Enhanced Recovery After Surgery (ERAS) pathways? Can J Anesth. 2015;62(2):120-30.
30. Neville A, Lee L, Antonescu I, Mayo NE, Vassiliou MC, Fried GM, et al. Systematic review of outcomes used to evaluate enhanced recovery after surgery. Br J Surg. 2014;101(3):159-170.
31. Chiu AK, Din N, Ai N. Standardising reported outcomes of surgery for intermittent exotropia--a systematic literature review." Strabismus. 2014;22(1):32-36.
32. Cook RJ, Heddle NM. Clinical trials evaluating pathogen-reduced platelet products: methodologic issues and recommendations. Transfusion. 2013;53(8):1843-1855.
