# Supplementary material for: Choosing Important Health Outcomes for Comparative Effectiveness Research: An Updated Review and User Survey
Source: PLoS One. 2016 Jan 19;11(1):e0146444. doi: 10.1371/journal.pone.0146444 (PMC4718543; doi:10.1371/journal.pone.0146444)
Supplement: S3 Table — (DOCX) [file pone.0146444.s004.docx]

**S3 Table.** Classification according to condition (n=227 published studies)

| **Disease Category** | **Disease Name** | **Number of studies** |
| --- | --- | --- |
| Anaesthesia & pain control (N=4) | Chronic pain | 1 |
|  | Chronic pain/ recurrent pain and acute pain | 1 |
|  | Post-operative nausea and vomiting | 1 |
|  | Chronic post-surgical pain after total knee replacement | 1 |
| Blood disorder (N=3) | Chronic graft-versus-host disease (GVHD) | 1 |
|  | Haemophilia and other bleeding disorders | 1 |
|  | Immune thrombocytopenic purpura | 1 |
| Cancer (N=37) | Acute myeloid leukemia | 1 |
|  | Rectal Cancer | 1 |
|  | Bone metastases | 1 |
|  | Breast cancer related lymphedema (BCRL) | 1 |
|  | Solid tumors | 1 |
|  | Colorectal cancer | 2 |
|  | Hepatocellular carcinoma | 1 |
|  | Human papillomavirus (Cervical cancer) | 1 |
|  | Localized prostate cancer | 1 |
|  | Advanced non-small-cell lung cancer | 2 |
|  | Leukemia | 1 |
|  | Systemic light-chain amyloidosis | 1 |
|  | Chemotherapy-induced nausea and vomiting | 2 |
|  | malignant lymphoma/non-Hodgkin's lymphoma | 1 |
|  | Hodgkin's disease and lymphoma | 1 |
|  | neuroendocrine tumors | 1 |
|  | Cancer (not specified types) | 1 |
|  | Cancer/Malignant disease | 3 |
|  | Head and neck cancer | 4 |
|  | Prostate cancer | 5 |
|  | Oral Mucositis (OM) | 1 |
|  | Ovarian Cancer | 2 |
|  | Myeloma | 1 |
|  | Anal cancer | 1 |
| Dentistry & oral health (N=12) | Supragingival dental plaque and gingivitis | 1 |
|  | Plaque and gingivitis | 1 |
|  | Caries | 1 |
|  | Chronic periodontitis (Anterior teeth) | 1 |
|  | Chronic periodontitis (Posterior teeth) | 1 |
|  | Edentulous/missing teeth | 3 |
|  | Extensive tooth decay | 1 |
|  | Implants in regenerated bone | 1 |
|  | Periodontitis | 2 |
| Endocrine & metabolic (N=3) | Obesity | 1 |
|  | Thyroid Eye Disease | 1 |
|  | Nonalcoholic steatohepatitis | 1 |
| Gastroenterology (n=8) | Chronic Hepatitis C | 1 |
|  | Hepatic encephalopathy | 1 |
|  | Crohn's disease | 1 |
|  | Nonvariceal upper gastrointestinal bleeding | 1 |
|  | Gastro-oesophageal reflux disease (GERD) | 1 |
|  | Irritable Bowel Syndrome | 1 |
|  | Antibiotic associated colitis, also known as clostridium difficile colitis, AKA pseudomembranous colitis | 1 |
|  | Inflammatory bowel disease (IBD) | 1 |
| Genetic disorders (N=2) | Cystic Fibrosis | 1 |
|  | Neurofibromatosis | 1 |
| Gynaecology (N=6) | Endometriosis-related pain | 1 |
|  | Abnormal uterine bleeding | 1 |
|  | Deeply infiltrative endometriosis (DIE) | 1 |
|  | Uterine Fibroids | 1 |
|  | Female sexual dysfunction | 2 |
| Health care of older people (N=2) | Sarcopenia | 1 |
|  | Dyspnoea or Breathlessness in Palliative Care | 1 |
| Heart & circulation (N=24) | Cardiac Arrest | 1 |
|  | Atherosclerosis | 1 |
|  | Chronic leg edema | 1 |
|  | Obstructive disease of supra-aortic arteries | 1 |
|  | Coronary heart disease | 1 |
|  | Pulmonary arterial hypertension | 1 |
|  | Pulmonary arterial hypertension related Systemic Sclerosis | 1 |
|  | Acute coronary syndrome | 1 |
|  | Acute heart failure syndromes (AKA Acute decompensated heart failure) | 1 |
|  | Atrial fibrillation | 2 |
|  | Aortic valve stenosis (AS)/Aortic stenosis (AS);Valvular heart disease | 2 |
|  | Cardiovascular disease | 1 |
|  | Obstructive coronary artery disease | 1 |
|  | Ischemic heart disease | 1 |
|  | Peripheral arterial occlusive disease(PAOD) | 1 |
|  | Deep venous thrombosis and pulmonary embolism | 1 |
|  | Critical limb ischemia | 1 |
|  | Acute stroke put with cardiovascular | 2 |
|  | Acute ischemic stroke likewise | 1 |
|  | Heart failure | 1 |
|  | Aortic dissection | 1 |
| Infectious disease (N=12) | Sepsis and Critical care (one for children) | 2 |
|  | Herpes Zoster | 1 |
|  | HIV | 1 |
|  | Hospital-acquired pneumonia and ventilator-associated pneumonia | 1 |
|  | Community-acquired pneumonia | 2 |
|  | Influenza | 1 |
|  | Leprosy | 1 |
|  | Malaria | 1 |
|  | Acute bacterial meningitis | 1 |
|  | Intraabdominal infection | 1 |
| Kidney disease (N=3) | Acute renal failure | 1 |
|  | Acute kidney injury | 2 |
| Lungs & airways (N=10) | Respiratory distress | 1 |
|  | Respiratory allergy | 1 |
|  | Asthma | 4 |
|  | COPD (Chronic Obstructive Pulmonary Disorder) | 1 |
|  | Chronic bronchitis and COPD | 1 |
|  | Connective tissue disease associated interstitial lung disease (CTD-ILD) and idiopathic pulmonary fibrosis (IPF) | 1 |
|  | Acute lung injury (ALI) | 1 |
| Mental health (N=3) | Bipolar disorder | 1 |
|  | Major Depressive Disorder | 1 |
|  | Forensic mental health | 1 |
| Neonatal care (N=3) | Neonatal apnea (also known as Apnea of prematurity, and Apnoea) | 1 |
|  | Neonatal cardiovascular instability | 1 |
|  | Neonatal seizures | 1 |
| Neurology (N=25) | Epilepsy | 1 |
|  | Infantile spasms  West syndrome (a form of Epilepsy) | 1 |
|  | Seizures | 1 |
|  | Headache | 1 |
|  | Parkinson's disease | 1 |
|  | Alzheimer's disease | 1 |
|  | Dementia | 2 |
|  | Intracranial cerebral atherosclerosis | 1 |
|  | Multiple Sclerosis (one for children) | 2 |
|  | Migraine | 2 |
|  | Cluster headache | 1 |
|  | Tension type headache | 1 |
|  | Amyotrophic lateral sclerosis/motor neurone disease | 2 |
|  | Hypoxic-ischemic brain injury | 1 |
|  | Traumatic brain injury | 1 |
|  | Insomnia (children) | 1 |
|  | Charcot-Marie-Tooth disease type 1A (CMT1A) | 1 |
|  | Chronic Inflammatory Demyelinating Polyradiculoneuropathy and Multifocal and Motor Neuropathy | 1 |
|  | Peripheral neuropathy | 1 |
|  | Cerebral palsy | 1 |
|  | Unilateral cerebral palsy | 1 |
| Orthopaedics & trauma (N=14) | Burns | 1 |
|  | Muskuloskeletal pain (subacute and chronic) | 1 |
|  | Fall Injury | 1 |
|  | Hip fracture | 2 |
|  | Osteoporosis | 1 |
|  | Low back pain | 1 |
|  | Acute low back pain | 1 |
|  | Spinal Cord Injury | 1 |
|  | Spinal disorders | 1 |
|  | Head Injury | 1 |
|  | ACL Injury | 1 |
|  | Distal radius fractures | 1 |
|  | Dupuytren's disease | 1 |
| Pregnancy & childbirth (N=4) | Maternity care | 1 |
|  | Gestational diabetes mellitus | 1 |
|  | Pre-eclampsia | 1 |
|  | Breech presentation | 1 |
| Rheumatology (N=31) | Systemic Lupus Erythematosus | 3 |
|  | Systemic Sclerosis | 2 |
|  | Systemic Sclerosis - related Arthritis | 1 |
|  | Systemic Sclerosis-associated Interstitial Lung Disease | 1 |
|  | Small-vessel Vasculitis/ANCA-associated Vasculitis | 1 |
|  | Systemic Vasculitis, AAV, anti-neutrophil cytoplasmic antibody-associated vasculitis; WG, Wegener’s granulomatosis | 1 |
|  | Osteoarthritis | 1 |
|  | Rheumatoid Arthritis | 6 |
|  | Psoriatic Arthritis | 1 |
|  | Idiopathic inflammatory myopathies (IIM) | 1 |
|  | Ankylosing spondylitis | 1 |
|  | Rheumatic diseases | 3 |
|  | Fibromyalgia syndrome | 2 |
|  | Gout | 1 |
|  | Arthritis | 1 |
|  | Juvenile systemic lupus erythematosus and juvenile dermatomyositis | 1 |
|  | Knee,hip and hand osteoarthritis | 1 |
|  | Sjögren's syndrome | 1 |
|  | Idiopathic arthritis-associated uveitis (juvenile) | 1 |
|  | Hand osteoarthritis | 1 |
| Skin (N=4) | Vitiligo | 1 |
|  | Eczema | 1 |
|  | Cutaneous leishmaniasis | 1 |
|  | Vulval skin condition | 1 |
| Tobacco, drugs, & alcohol dependence (N=4) | Drug dependence | 1 |
|  | Addiction (substance) | 1 |
|  | Nicotine, alcohol and cocaine abuse/dependence | 1 |
|  | Addiction (gambling) | 1 |
| Urology (N=4) | Peyronie's disease | 1 |
|  | Nocturnal Enuresis | 1 |
|  | Pelvic organ prolapse | 1 |
|  | Male sexual dysfunction/disorders | 1 |
| Wounds (N=2) | Non-healing wounds | 1 |
|  | Venous leg ulcers | 1 |
| Others (N=7) |  |  |
| Benign disease | Benign/ Non-malignant diseases | 1 |
| Chronic conditions | JIA, OI, Achondroplasia, Hemophilia, Cerebral Palsy, Spina Bifida, CF, Cancer | 1 |
| Ear, Nose & Throat | Tinnitus | 1 |
| Eyes & vision | Intermittent exotropia | 1 |
| Intensive care | Critical illness/ ICU disease | 1 |
| Rehabilitation | Recovery after surgery | 1 |
| N/A | N/A – Platelet transfusion trials | 1 |
| TOTAL |  | **227** |
