# Supplementary material for: Choosing Important Health Outcomes for Comparative Effectiveness Research: An Updated Review and User Survey
Source: PLoS One. 2016 Jan 19;11(1):e0146444. doi: 10.1371/journal.pone.0146444 (PMC4718543; doi:10.1371/journal.pone.0146444)
Supplement: S4 Table — (DOCX) [file pone.0146444.s005.docx]

**S4 Table.** Participant groups involved in selecting outcomes in new studies identified in the review update (n=29)

| **Participants category (total number of studies involving this particular participant category)** | **Sub-category (not mutually exclusive)** | **n (Frequency of the sub-category participants)** |
| --- | --- | --- |
| **Clinical experts (n = 21/22)** | Clinical experts | 15 |
|  | Clinical research expertise | 10 |
|  | Clinical trialists/Members of a clinical trial network | 2 |
| **Public representatives (n= 13/22)** | Patients | 11 |
|  | Carers | 1 |
|  | Patient support group representatives | 1 |
| **Non-clinical research experts (n = 10/22)** | Researchers | 4 |
|  | Statisticians | 4 |
|  | Epidemiologists | 2 |
|  | Methodologists | 4 |
| **Authorities (n = 5/22)** | Regulatory agency representatives | 4 |
|  | Governmental agencies | 1 |
|  | Policy makers | 1 |
| **Industry representatives (n = 4/22)** | Pharmaceutical industry representatives | 3 |
|  | Device manufacturers | 1 |
| **Others (n = 2/22)** | Funding bodies | 1 |
|  | Yoga therapists/instructors | 1 |
| **No details given (n= 7/29)** |  |  |
